# Interfacial Crosslinking for Efficient and Stable Planar TiO_2_ Perovskite Solar Cells

**DOI:** 10.1002/advs.202402796

**Published:** 2024-07-03

**Authors:** Linrui Duan, Siyu Liu, Xiaobing Wang, Zhuang Zhang, Jingshan Luo

**Affiliations:** ^1^ Institute of Photoelectronic Thin Film Devices and Technology State Key Laboratory of Photovoltaic Materials and Cells Tianjin Key Laboratory of Efficient Solar Energy Utilization Ministry of Education Engineering Research Center of Thin Film Photoelectronic Technology Nankai University Tianjin 300350 China; ^2^ Frontiers Science Center for New Organic Matter Nankai University Tianjin 300071 China; ^3^ Haihe Laboratory of Sustainable Chemical Transformations Tianjin 300192 China

**Keywords:** electron transport layer, interface modification, perovskite layer, perovskite solar cell

## Abstract

The buried interface between the electron transport layer (ETL) and the perovskite layer plays a crucial role in enhancing the power conversion efficiency (PCE) and stability of n–i–p type perovskite solar cells (PSCs). In this study, the interface between the chemical bath deposited (CBD) titanium oxide (TiO_2_) ETL and the perovskite layer using multi‐functional potassium trifluoromethyl sulfonate (SK) is modified. Structural and elemental analyses reveal that the trifluoromethyl sulfonate serves as a crosslinker between the TiO_2_ and the perovskite layer, thus improving the adhesion of the perovskite to the TiO_2_ ETL through strong bonding of the ─CF_3_ and ─SO_3_^−^ terminal groups. Furthermore, the multi‐functional modifiers reduced interface defects and suppressed carrier recombination in the PSCs. Consequently, devices with a champion PCE of 25.22% and a fill factor (FF) close to 85% is achieved, marking the highest PCE and FF observed for PSCs based on CBD TiO_2_. The unencapsulated device maintained 81.3% of its initial PCE after operating for 1000 h.

## Graphical abstract

This study introduces a multifunctional potassium trifluoromethyl sulfonate (SK) to modify the chemical bath deposited titanium oxide (TiO_2_) electron transport layer and perovskite interface. The modification enhanced the perovskite crystallinity and reduced the non‐radiative carrier recombination in perovskite solar cells. As a result, the TiO_2_‐SK significantly improves the power conversion efficiency (PCE) of FAPbI_3_ from 24.01% to 25.22%.

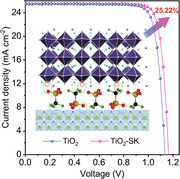

## 1 Introduction

Organic–inorganic hybrid perovskite solar cells (PSCs) have garnered considerable attention in the photovoltaic industry due to their cost‐effective production and outstanding optoelectronic properties.^[^ ^1^, ^2^, ^3^, ^4^, ^5^ ^]^ Over the past decade, PSC technology has achieved a remarkable breakthrough in power conversion efficiency (PCE) of 26.1%, comparable to the highest‐performing single‐crystalline solar cells. These advancements position PSCs as having significant commercial potential. However, obstacles to PSC commercialization persist, such as stability and hysteresis effects.^[^ ^6^, ^7^, ^8^, ^9^, ^10^, ^11^, ^12^, ^13^ ^]^

In the general n‐i‐p structure, titanium oxide (TiO_2_) is commonly used as the electron transport layer (ETL) due to its suitable band energy level, low cost, and good thermal stability. The buried interfacial contact between the perovskite layer and ETL is crucial for the performance of PSCs.^[^ ^14^, ^15^, ^16^, ^17^ ^]^ Photovoltaic performance parameters are closely linked to the interfacial charge transfer and transport ability. Efficient charge transfer and transport can lead to improved open circuit voltage (*V*_oc_), short circuit current density (*J*_sc_), and fill factor (*FF*).^[^ ^18^, ^19^, ^20^ ^]^ However, these processes will be significantly compromised in the presence of defects at the interfaces. Generally, the perovskite film surface contains a higher defect density compared to the bulk perovskite film, including uncoordinated Pb^2+^, I^−^ and anti‐site defects Pb_I and_ I_Pb_.^[^ ^12^, ^21^ ^]^ As for the TiO_2_ ETL, there are oxygen and titanium vacancies, as well as anti‐site defects (Ti_O_ and O_Ti_). All these defects at the heterojunction interface can create shallow and deep defect levels in the perovskite film and ETL, acting as recombination centers and leading to non‐radiative recombination in PSCs.^[^ ^22^ ^]^ In addition to impacting photovoltaic performance, these defects also influence the stability of PSCs. Perovskite crystals tend to collapse at defect sites, so reducing the defect density improves the stability of the perovskite film. Moreover, high electronic trap states in TiO_2_ ETL trigger undesirable charge accumulation and recombination, resulting in low PCE and significant hysteresis. Furthermore, O vacancies increase the catalytic activity of TiO_2_, leading to UV light instability and limiting the operational device lifetime.^[^ ^23^, ^24^ ^]^

Therefore, numerous researchers have concentrated on passivating interfacial defects by introducing functional materials. Modifying the surface of TiO_2_ is simple and effective in enhancing electron transport capability and adjusting energy level alignments, thereby improving device performance.^[^ ^1^, ^9^, ^14^, ^17^, ^25^ ^]^ For example, Tan et al. introduced PbCl_2_ to modify TiO_2_ surface. The resulting Cl‐capped TiO_2_ ETL displays suppressed anti‐site defects, leading to reduced interfacial recombination. Consequently, the PSCs exhibit a power PCE of 21.4% with negligible hysteresis.^[^ ^26^ ^]^ Kim et al. utilized Li‐doped mesoporous TiO_2_ with Li_2_CO_3_ to enhance electrical properties, resulting in an improved PCE from 23.15% to 25.28%.^[^ ^27^ ^]^ The introduction of Li salts reduced defects and optimized energy level alignments, benefiting carrier extraction and transport, consequently improving *V*_oc_ and *J*_sc_. Additionally, Li et al. introduced organic ligands to regulate the crystal growth of TiO_2_ ETL through chemical bath deposition (CBD). These ligands also passivated the TiO_2_ surface, leading to an improved PCE of 24.8%.^[^ ^17^ ^]^ These studies underscore the significance of interfacial engineering in developing efficient PSCs.

In this study, we have developed a strategy that involves careful chemical selection to reduce interfacial defects in both the ETL and the perovskite layer using a cross‐linker. We chose potassium trifluoromethyl sulfonate (SK) to modify the chemical bath deposited TiO_2_ ETL. This strategy offers several potential benefits. First, the modifier at the interface enhances the adhesion of the perovskite with the oxide substrate through its two functional groups, resulting in a stable interfacial contact. Second, the ─CF_3_ and ─SO_3_^−^ groups could passivate the defects in the TiO_2_ ETL and perovskite film, respectively, which is advantageous for improving the photovoltaic performance of PSCs. Third, the K^+^ cation can also act as a growth‐controlling agent, leading to the formation of a uniform film with large crystal grains. The combined synergistic effects of these three factors contribute to improved PCE and long‐term stability of the device. Our work presents a new and general strategy for preparing high‐efficiency PSCs.

## 2 Result and Discussion

**Figure 1:**
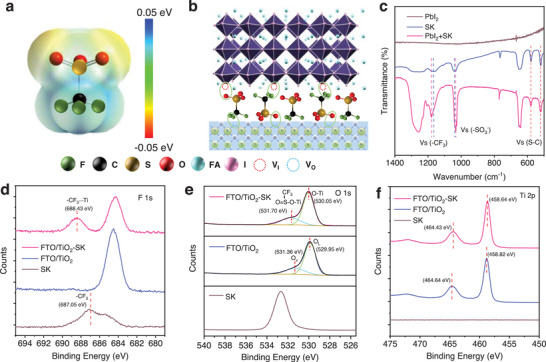
a) The electrostatic potential (ESP) distributions of potassium trifluoromethyl sulfonate (SK). b) The schematic diagram of the cross‐linking between the TiO_2_ electron transport layers (ETL) and perovskite layer. c) Fourier transform infrared spectroscopy (FTIR) tests of the PbI_2_, SK, and SK‐PbI_2_ mixture. d–f) X‐ray photoelectron spectroscopy (XPS) tests of the SK, control TiO_2_ and SK treated TiO_2_ (TiO_2_‐SK) ETLs.

We deposited planar TiO_2_ as the ETL and treated it with a SK solution. The SK modification increased the wettability of the TiO_2_ as shown in Figure S1 (Supporting Information). The UV–vis absorption spectra and tauc‐plot curves are presented in Figure S2 (Supporting Information), corresponding to a bandgap of 3.015 eV for the control TiO_2_ and TiO_2_‐SK ETLs. The formamidinium lead iodide (FAPbI_3_) PSCs were fabricated using a one‐step method, and further details are presented in the experimental section. **Figure** **1**a illustrates the electrostatic potential (ESP) distributions of the SK modifier. The SK agent exhibits distinctive positive and negative surface potential on the two sides of the molecule. Figure 1b shows a schematic diagram of the PSCs based on TiO_2_ ETL with the SK modifier. To comprehend the function of the SK modifier at the interface, we conducted Fourier transform infrared spectroscopy (FTIR) tests of the PbI_2_, SK, and PbI_2_‐SK mixtures (Figure 1c; Figure S3, Supporting Information). The results reveal that the stretching vibration of S═O is located at 1039.31 cm^−1^ for the SK sample, while the value shifted to 1033.10 cm^−1^ for the PbI_2_‐SK sample. Additionally, the stretching vibration of ─CF_3_ is located at 1165.44 cm^−1^ for the SK sample and shifted to 1179 cm^−1^. We further carried out the X‐ray photoelectron spectroscopy (XPS) of FAPbI_3_ and SK modified FAPbI_3_ perovskite films. The binding energy of F 1s increased from 687.05 to 688.70 eV while the binding energy of Pb 4f_5/2_ and Pb 4f_7/2_ reduced from 143.39 and 138.53 eV to 143.29 and 138.41 eV, respectively, as shown in Figure S4 (Supporting Information), indicating the interaction between the SK modifier and perovskite films. Due to the electron‐donating ability of the ─CF_3_ unit, the electron cloud density of F will decrease while the electron cloud density of Pb increases after accepting electrons from the ─CF_3_ units. Therefore, the binding energy of F inner shell electrons increased because of the weakened shielding effect of the outer electrons, leading to an increased binding energy of F 1s and a reduced binding energy of Pb 4f. Consequently, the ─SO_3_^−^ and ─CF_3_ units could act as Lewis‐base and tend to coordinate with the uncoordinated Pb^2+^, reducing defects in the perovskite film and charge recombination of the PSCs.^[^ ^15^ ^]^

To investigate the interaction between the SK and TiO_2_ ETL, we performed XPS tests of the control TiO_2_ and SK treated TiO_2_ (TiO_2_‐SK) (Figure 1d–f; Figure S5, Supporting Information). Figure 1d shows the F 1s XPS spectrum, with the peak located at 687.05 eV for ─CF_3_ in the SK modifier. Due to the electron‐donating ability of ─CF_3_, the electron cloud density decreased after the interaction between the ─CF_3_ and TiO_2_, resulting in the increased binding energy of F 1s to 688.43 eV. In contrast, the electron density of Ti^4+^ increased after accepting electrons from ─CF_3_, leading to a slight decrease in the binding energy for Ti 2p^1/2^ and Ti 2p^3/2^ from 464.64 and 458.82 eV to 464.43 and 458.64 eV, respectively, as shown in Figure 1e. Figure 1f shows that the binding energy of O 1s for the SK sample is located at 532.67 eV. Meanwhile, the TiO_2_ ETL could be decoupled into two peaks, which are ascribed to the lattice oxygen (O_L_) and oxygen vacancy (O_V_), corresponding to a binding energy of O 1s at 529.95 and 531.36 eV.^[^ ^28^ ^]^ For TiO_2_‐SK ETL, the two peaks were shifted to 530.05 and 531.70 eV due to the occupation of the O_V_ by the O atom of the ─SO_3_^−^ unit. Overall, both the aggregative negative potential of ─CF_3_ and the positive potential of ─SO_3_^−^ could coordinate with the vacancy in the TiO_2_ ETLs and perovskite layers due to its electron donating ability. As a result, the functional ─SO_3_^−^ and ─CF_3_ units of SK agents could modify the perovskite and TiO_2_ layer, acting as a crosslinker at the interface to improve the interfacial contact of the PSCs.

**Figure 2:**
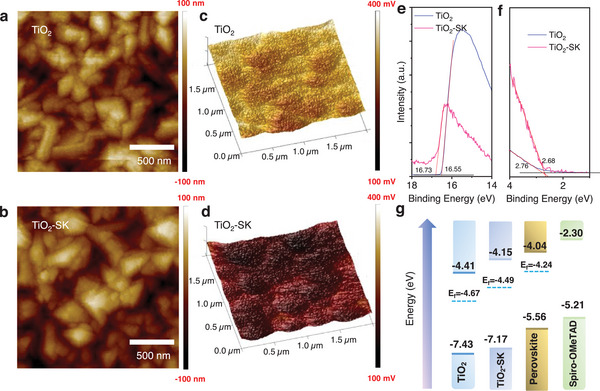
a,b) Atomic force microscopy (AFM) images of the TiO_2_ and TiO_2_‐SK ETL substrates. c,d) Kelvin probe force microscopy (KPFM) tests of the TiO_2_ and TiO_2_‐SK ETL substrates (scanning area: 2 µm ×2 µm). e,f) Ultraviolet photoelectron spectroscopy (UPS) tests of the TiO_2_ and TiO_2_‐SK ETL substrates. g) Energy level alignments of the different layers in perovskite solar cells (PSCs).

We further investigated the surface morphology of the TiO_2_ and TiO_2_‐SK ETLs using atomic force microscopy (AFM) and scanning electron microscopy (SEM) shown in Figure S6 (Supporting Information). A thin and compact TiO_2_ layer (≈30 nm) is deposited on the FTO substrates. The AFM results show that the root mean square roughness (RMS) of the TiO_2_ and TiO_2_‐SK ETLs is 37.7 and 32.9 nm, respectively, as depicted in **Figure** **2**a,b. Additionally, we utilized Kelvin probe force microscopy (KPFM) to measure the contact potential difference (CPD = V_tip_ − V_sample_). The CPD images are shown in Figure 2c,d, and the CPD value of the TiO_2_ film decreased from 285 to 186 mV. These results indicate that TiO_2_‐SK ETL displays a reduced work function and an increased Fermi level. The strong interaction of SK with TiO_2_ alters the energy levels of the TiO_2_ ETL. To further assess the energy levels, ultraviolet photoelectron spectroscopy (UPS) tests were conducted (Figure S7 Supporting Information). The E_cutoff_ edge of the UPS spectra of the TiO_2_ shifted from 16.55 to 16.73 eV after modification with SK, as illustrated in Figure 2e,f, resulting in an elevated Fermi level from −4.67 to −4.49 eV, closer to that of the FAPbI_3_ film (E_fermi_ = −4.24 eV). Consequently, the conduction band minimum (CBM) for TiO_2_ and TiO_2_‐SK were calculated to be −4.41 and −4.15 eV, respectively, which is beneficial for the energy level alignments between the ETL and perovskite film (CBM = −4.04 eV). Figure 2g illustrates the energy level alignments of the PSCs. The decreased energy level offset between the TiO_2_‐SK ETL and FAPbI_3_ perovskite film is advantageous for interfacial charge extraction and transfer, contributing to improved photovoltaic performance.

**Figure 3:**
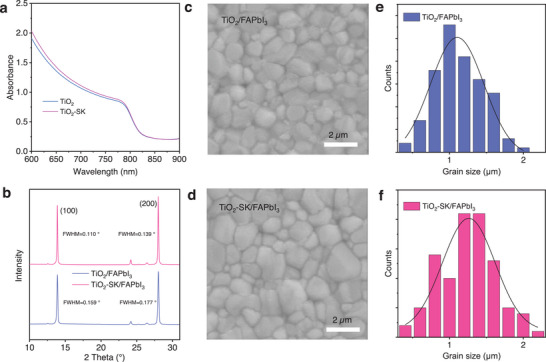
a) UV–vis absorption spectra of the TiO_2_/FAPbI_3_ and TiO_2_‐SK/FAPbI_3_ films. b) X‐ray diffraction (XRD) patterns of the TiO_2_/FAPbI_3_ and TiO_2_‐SK/FAPbI_3_ films. c,d) canning electron microscope (SEM) images of the TiO_2_/FAPbI_3_ and TiO_2_‐SK/FAPbI_3_ films. e,f) Crystal grain size distributions of the TiO_2_/FAPbI_3_ and TiO_2_‐SK/FAPbI_3_ films.

We then investigated the properties of the perovskite films. The FAPbI_3_ film, when using reference TiO_2_ and TiO_2_‐SK ETLs, was designated as TiO_2_/FAPbI_3_ and TiO_2_‐SK/FAPbI_3_, respectively. Both the TiO_2_/FAPbI_3_ and TiO_2_‐SK/FAPbI_3_ perovskite films exhibit the same absorption onset at 811 nm, corresponding to an optical bandgap of 1.52 eV, **Figures** **3**a and S8 (Supporting Information). The TiO_2_‐SK/FAPbI_3_ perovskite film shows a slightly higher absorption coefficient compared to the TiO_2_/FAPbI_3_ perovskite film. The X‐ray diffraction patterns (XRD) of the TiO_2_/FAPbI_3_ and TiO_2_‐SK/FAPbI_3_ perovskite films reveal peaks at 13.92° (100) and 28.01° (200), as shown in Figure 3b. The full width at half maximum (FWHM) of the TiO_2_/FAPbI_3_ are 0.159 and 0.177, which is larger than that of 0.110 and 0.139 for TiO_2_‐SK/FAPbI_3_ perovskite films shown in Figure S9 (Supporting Information). The higher intensity of the characteristic peaks and smaller FWHM for TiO_2_‐SK/FAPbI_3_ perovskite film indicates better crystallinity. SEM was employed to investigate the morphology of perovskite films. From SEM images in Figures 3c,d and S10 (Supporting Information), it is evident that the TiO_2_‐SK/FAPbI_3_ perovskite film displays larger crystal grains compared to the TiO_2_/FAPbI_3_ perovskite film, mainly attributed to the K^+^ diffusion into the FAPbI_3_ film, promoting nucleation crystallization growth and enlarge the crystal size.^[^ ^14^, ^25^ ^]^ The average grain size for the TiO_2_‐SK/FAPbI_3_ perovskite film is 1.3 µm, larger than the 1.1 µm for the TiO_2_/FAPbI_3_ perovskite film, as shown in Figure 3e,f. The increased grain size results in fewer defects, as the defects are generally distributed on the grain boundaries of the perovskite films.^[^ ^5^ ^]^ The improved morphology contributes to the enhancement of the photovoltaic performance of the PSCs.

**Figure 4:**
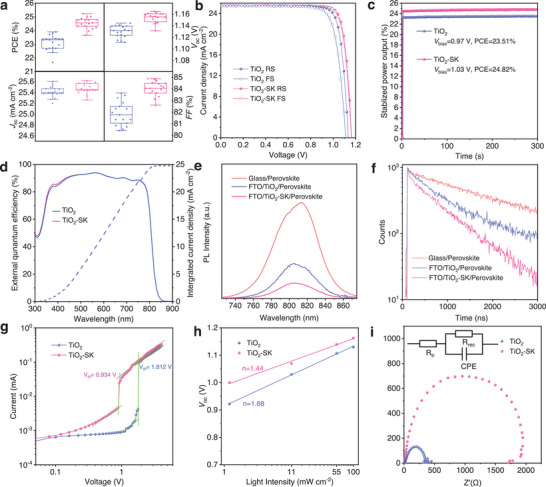
a) Photovoltaic performance of the TiO_2_‐FAPbI_3_ and TiO_2_‐SK/FAPbI_3_ PSCs. b) *J–V* curves of the TiO_2_‐FAPbI_3_ and TiO_2_‐SK/FAPbI_3_ PSCs. c) Stabilized power output (SPO) of the TiO_2_‐FAPbI_3_ and TiO_2_‐SK/FAPbI_3_ PSCs. d) External quantum efficiency (EQE) test of the TiO_2_‐FAPbI_3_ and TiO_2_‐SK/FAPbI_3_ PSCs. e,f) Photoluminescence and time‐resolved photoluminescence (TRPL) tests of the TiO_2_/FAPbI_3_ and TiO_2_‐SK/FAPbI_3_ films. g) Space‐charged limited current (SCLC) of the device in a structure of FTO/TiO_2_/perovskite/PCBM/BCP/Au. h) The dependence of *V*_oc_ on the light intensity of the TiO_2_‐FAPbI_3_ and TiO_2_‐SK/FAPbI_3_ PSCs. i) Electrical impedance spectroscopy (EIS) measurements of the TiO_2_‐FAPbI_3_ and TiO_2_‐SK/FAPbI_3_ PSCs.

To investigate the photovoltaic performance, we fabricated FAPbI_3_ PSCs using a device configuration of FTO/c‐TiO_2_/perovskite/Spiro‐OMeTAD/Au, and the experimental details are provided in the Experimental Section. **Figures** **4**a and S11 (Supporting Information) illustrate the photovoltaic performance of the TiO_2_‐SK PSCs compared to that of the control TiO_2_ PSCs, with detailed parameters displayed in Table S1 (Supporting Information). Notably, significant improvements are observed in the *V*_oc_ and *FF*, which improved from 1.13 ± 0.05 V to 1.16 ± 0.05 V and 82.5 ± 1.0% to 84.0 ± 0.9%, respectively. Additionally, Figure 4b presents the current density–voltage (*J–V*) curves of the champion devices. The TiO_2_‐SK PSC exhibits a remarkable reverse scanning PCE of 25.22% with a *V*_oc_ of 1.165 V, a *J*_sc_ of 25.51 mA cm^−2^ and an *FF* of 84.9%. The forward scanning PCE is 24.80% with a *V*_oc_ of 1.161 V, a *J*_sc_ of 25.50, and an *FF* of 83.8%. Due to enhanced carrier extraction ability and suppressed nonradiative recombination, the TiO_2_‐SK PSC displays less hysteresis. In contrast, the control PSC shows a reverse scanning PCE of 24.01% with a *V*_oc_ of 1.130 V, a *J*_sc_ of 25.47 mA cm^−2^ and an *FF* of 83.5%, while the forward scanning PCE is 22.04% with a *V*_oc_ of 1.11 V and a *J*_sc_ of 25.44 mA cm^−2^ and an *FF* of 78.1%. Furthermore, Figure 4c displays the stabilized power outputs (SPO) of the TiO_2_ and TiO_2_‐SK PSCs, corresponding to the PCEs of 23.51% and 24.82%. External quantum efficiency (EQE) measurements were performed to ascertain the *J*_sc_ value, resulting in integrated *J*_sc_ values of 24.85 mA cm^−2^ for the control TiO_2_ PSC and 24.91 mA cm^−2^ for the TiO_2_‐SK PSC, as shown in Figure 4d.

To investigate the carrier extraction and transport ability of the TiO_2_ and TiO_2_‐SK, we conducted steady‐state photoluminescence (PL) and time‐resolved photoluminescence (TRPL) tests. The PL results indicate an emission peak at ≈ 810 nm, as shown in Figure 4e,f, and the FAPbI_3_‐SK film displays lower PL intensity compared to the FAPbI_3_ film due to enhanced charge transfer from the perovskite layer to the TiO_2_ ETL. This result was further confirmed by the TRPL test, which revealed faster PL quenching for TiO_2_‐SK/FAPbI_3_ film, verifying a shorter carrier lifetime of 761.7 ns compared to 1142.6 ns for the TiO_2_/FAPbI_3_ film (Table S2 Supporting Information). The reduced lifetime indicates efficient electron extraction at the TiO_2_‐SK ETL/perovskite interface with SK modification, thereby reducing charge accumulation at the interface and suppressing device hysteresis. To quantify the trap defect density of the perovskite films, we conducted space‐charge limited current (SCLC) tests on the electron‐only devices in a structure of FTO/TiO_2_/Perovskite/PCBM/BCP/Au (Figure 4g). The V_tfl_ values were estimated to be 0.934 and 1.812 V, respectively. The trap density was calculated using the equation N_trap_ = 2ɛ_r_ɛ_0_V_TFL_/eL^2^, where e represents the elementary charge, and ɛ_r_ and ɛ_0_ are the relative dielectric constant and vacuum permittivity, respectively.^[^ ^29^, ^30^ ^]^ The trap densities of the TiO_2_ and TiO_2_‐SK PSCs were estimated to be 2.96 × 10^16^ and 1.51 × 10^16^.^[^ ^31^ ^]^

The relationship between *V*_oc_ and light intensity was also analyzed, as depicted in Figure 4h. Linear fitting of *V*_oc_ versus log‐scaled light intensity allows the reflection of trap‐assisted recombination through the slope value and nkT/q (where k represents the Boltzmann constant, T is temperature, q is the electric charge, and n is the ideality factor). The smaller ideality factor of 1.44 for the TiO_2_ PSC compared to 1.68 for the TiO_2_‐SK PSC indicates a suppressed trap‐assisted recombination. Furthermore, electrical impedance spectroscopy (EIS) measurements were conducted to further investigate the interfacial charge transport behavior in PSCs (Figure 4i; Table S3, Supporting Information). The increased recombination resistance (R_rec_ = 1898 Ω) for TiO_2_‐SK PSC indicates suppressed carrier recombination compared to the TiO_2_ PSC (R_rec_ = 349 Ω). Additionally, the decreased transport resistance (R_tr_) for the TiO_2_‐SK PSC (13.78 Ω vs 15.05 Ω for TiO_2_ PSC) is beneficial for the charge transport, resulting in an improved *FF* and reducing hysteresis.^[^ ^32^, ^33^ ^]^

**Figure 5:**
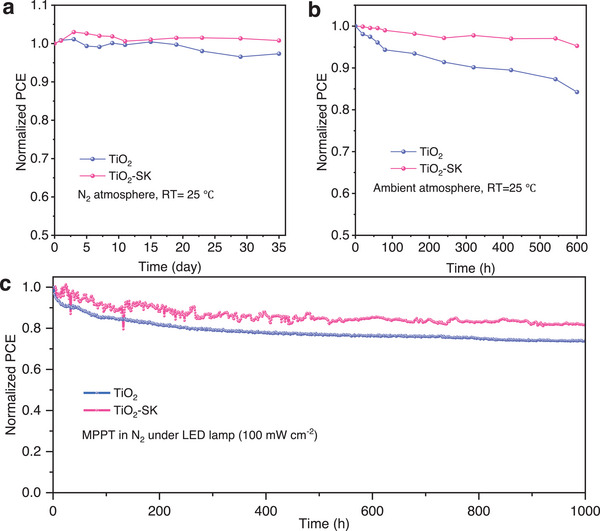
a) The stability of TiO_2_ and TiO_2_‐SK PSCs stored in N_2_ atmosphere. b) The stability of TiO_2_ and TiO_2_‐SK PSCs stored in ambient conditions (RT = 25 °C, RH = 20%). c) The maximum power point tracking (MPPT) tests of the TiO_2_ and TiO_2_‐SK PSCs in N_2_ under an LED lamp (light intensity 100 mW cm^−2^).

We assessed the stability of the PSCs by storing them in an N_2_ atmosphere, and the results are presented in **Figures** **5**a and S12 (Supporting Information). The TiO_2_‐SK PSC retained almost 100% of their initial PCE for 35 days, while the control device decreased to 97%. Additionally, we tested the stability of the unencapsulated PSCs in ambient conditions (RH = 20 ± 5%, RT = 25 ± 5 °C), and found that the TiO_2_ and TiO_2_‐SK PSCs preserved 84% and 95% of the initial efficiency, respectively, as shown in Figure 5b and Figure S13 (Supporting Information). Furthermore, we investigated the operational stability of the PSCs by aging the unencapsulated devices under a nitrogen atmosphere, using maximum power point (MPP) tracking under an LED lamp (light intensity 100 mw cm^−2^). Figure 5c shows that the normalized PCE of the TiO_2_ PSC decreased to 73.7%, while the TiO_2_‐SK PSC remained at 81.3% of the initial efficiency after operating for 1000 h. The improved stability of the TiO_2_‐SK PSCs is attributed to the reduced interfacial defect density and improved energy level alignments after interfacial cross‐linking modification. The reduction in recombination and charge accumulation during the charge transfer process contributes to the operational stability of the PSCs under light illumination.^[^ ^21^, ^34^, ^35^ ^]^ Moreover, the high crystallinity and enlarged grain size resulting from K‐doping also contribute to better stability and photovoltaic performance of the TiO_2_‐SK PSCs.

## 3 Conclusion

In summary, our introduction of multi‐functional SK modifier has led to improvements in both the power conversion efficiency and stability of the PSCs. The two terminal groups ─SO_3_^−^ and ─CF_3_ can interact with the TiO_2_ ETL and perovskite layer through ionic and hydrogen bonds, thereby strengthening the adhesion between the TiO_2_ ETL and perovskite layer. This interaction also reduces interfacial defects and minimizes the non‐radiative recombination of the PSCs. Furthermore, the SK modifier improves the morphology and enlarges the grain size of the perovskite film, which contributes to a reduced defect density and prolonged carrier lifetime of the perovskite film. In addition, the modification adjusts the energy level alignments of the TiO_2_ ETL and perovskite layer, which is beneficial for interfacial charge transfer, thereby reducing the charge accumulation at the TiO_2_/perovskite interface. As a result, the FAPbI_3_ PSCs display an improved *V*_oc_ and *FF* close to 85%. In conclusion, we achieved a champion PCE of 25.22% with negligible hysteresis and excellent operational stability. Our work provides a straightforward method to fabricate efficient and stable FAPbI_3_ PSCs.

## Conflict of Interest

The authors declare no conflict of interest.

## Author Contributions

J.L. designed the experiment and supervised the study. L.D. conducted the device fabrication, characterization, and manuscript preparation. S.L. participated in the SCLC and FTIR tests. X.W. contributed to the ESP analysis. Z.Z. conducted the EIS tests. L.D. and J.L. wrote the manuscript. All authors discussed the results and contributed to the manuscript revision.

## Supporting information

Supporting Information

Supporting Information

## Data Availability

The data that support the findings of this study are available from the corresponding author upon reasonable request.
