# Supplementary material for: Interfacial Crosslinking for Efficient and Stable Planar TiO2 Perovskite Solar Cells
Source: Adv Sci (Weinh). 2024 Jul 3;11(33):2402796. doi: 10.1002/advs.202402796 (PMC11434036; doi:10.1002/advs.202402796)
Supplement: Supplementary file 1 — Supporting Information [file ADVS-11-2402796-s001.docx]

**Supplementary Materials**

**Interfacial Crosslinking for Efficient and Stable Planar TiO_2_ Perovskite Solar Cells**

Linrui Duan^1^, Siyu Liu^1^, Xiaobing Wang^1^, Zhuang Zhang^1^, Jingshan Luo^1,2,3^*

^1^Institute of Photoelectronic Thin Film Devices and Technology, State Key Laboratory of Photovoltaic Materials and Cells, Tianjin Key Laboratory of Efficient Solar Energy Utilization, Ministry of Education Engineering Research Center of Thin Film Photoelectronic Technology, Nankai University, Tianjin 300350, China.

^2^Frontiers Science Center for New Organic Matter, Nankai University, Tianjin 300071, China

^3^Haihe Laboratory of Sustainable Chemical Transformations, Tianjin 300192, China

*Email: [jingshan.luo@nankai.edu.cn](mailto:jingshan.luo@nankai.edu.cn)

**Experimental Section**

**Materials**. Lead iodide (PbI_2_) is purchased from Alfa Aesar. Formamidinium iodide (FAI) and 2′,7,7′-Tetrakis[N,N-di(4-methoxyphenyl)amino]−9,9′-spirobifluorene (Spiro-OMeTAD)titanium, 4-tert-butyl pyridine (TBP) are purchased from Xi’an Polymer Light Technology Corp. Ultra-dry dimethylformamide (DMF), dimethyl sulfoxide (DMSO), ethanol (EtOH), and chlorobenzene (CB) are purchased from Acros., lithium bis(trifluorosulfonyl) imide (LiTFSI), Titanium chloride, and methylammonium chloride (MACl), potassium trifluoromethyl sulfonate (SK) are purchased from Sigma-Aldrich. Fluorine-doped tin oxide (FTO) (10 Ω/sq) conductive glass is purchased from Asahi. All the chemicals are used as received without further purification.

**TiO_2_ and TiO_2_-SK preparation.** FTO substrates are cleaned by using 2% Hellmanex aqueous solution, deionized water, acetone, and ethanol consecutively by sonicating for 15 min for each solvent. After drying with compressed N_2_, Plasma treatment for 5 min is applied for further cleaning. The chemical bath deposition (CBD) was prepared by adding 1 ml titanium chloride (TiCl_4_) dropwise into 100 ml ice water, 2 ml HCl (37%) was added before the TiCl_4_ addition to suppress the fast hydrolysis. Finally, a clean solution was obtained. The anti-corrosion tape was used to cover one edge and the back side of the FTO to prevent the TiO_2_ deposition. Briefly, the substrates are immersed into the TiCl_4_ precursor for 30 min at 70 ℃. After the deposition, the TiCl_4_ solution turns from clean to a little cloudy solution. The FTO/TiO_2_ substrates were washed with diluted water and heated at 100 ℃ for 30 min. The FTO/TiO_2_-SK substrates were prepared by spinning coating SK solution (1mg/mL in DI water) onto the TiO_2_ substrates at 3000 rpm for 30 s, followed by heating at 100 ℃ for 10 min. The substrates were plasma-treated before use.

**FAPbI_3_ perovskite film and device preparation.** The precursor was prepared by dissolving PbI_2_, FAI and MACl (35%mol) in DMF/DMSO (v/v=4:1) with a concentration of 1.8 M. The FAPbI_3_ perovskite layer is deposited using the antisolvent method. The perovskite precursor solution was spin-coated onto the FTO/TiO_2_ and FTO/TiO_2_-SK substrates at 5000 rpm for 50 s, 600 µL of diethyl ether was added after 10 s. After spin-coating, the substrates were annealed at 150 °C for 10 min, then 100 °C for 30 min. The whole procedure proceeded in an ambient condition RH=30%, RH =25 ℃. The perovskite films were then treated with OAI (2 mg/mL in IPA). The doped spiro-OMeTAD solution was spin-casted on the surface of the perovskite at 4000 rpm with an acceleration rate of 2000 rpm/s for 30 s. Spiro-OMeTAD is dissolved in chlorobenzene with a concentration of 90 mg/ml, which is doped by 23 μl LiTFSI (520mg/mL in CH_3_CN) and 39.5 μl 4-tert-butyl pyridine, and 10 μl FK209 (375mg/ mL in ACN). The device fabrication is completed with the deposition of gold electrode (~80 nm) by thermal evaporation.

**Photovoltaic performance measurements.** The prepared perovskite solar cells were measured by using a 300W Xenon light source from Newport. Before each measurement, the exact light intensity was determined using a calibrated Si reference diode equipped with an infrared cut-off filter (KG-5). Keithley 2400 is used for the current-voltage scan by applying an external voltage bias and measuring the response current with a scan rate of 20 mV from 1.4 V to -0.2 V (reverse scan) followed by a return scan with a delay time of 0.05 s. The device area was 0.24 cm^2^ (0.24 cm × 1 cm). The cells were masked with a black metal mask with an area of 0.0896 cm^2^. External quantum efficiency (EQE) was recorded with a commercial apparatus (Aekeo-Ariadne, Cicci Research s.r.l.) based on a 300W Xenon lamp. The stability data is acquired from MPP tracking of unencapsulated devices under a continuous nitrogen flow at 25 °C.

**PL, TRPL, and UV-*vis* measurements.** UV-*vis* absorptions were measured using Varian Cary 500 spectrometer (Varian USA). Steady-state photoluminescence (PL) and time-resolved photoluminescence (TRPL) spectra were recorded by a fluorescence spectrometer (FLS980, Edinburgh Instruments, UK). The light was illuminated from the glass side of the perovskite film, and the excitation source is a laser diode with a wavelength of 450 nm. For glass/FAPbI_3_ film, the carrier lifetime *t* was calculated by mono-exponential fitting according to y = A1*exp(-x/*t*_1_) + y0, while the FTO/TiO_2_/FAPbI_3_ and FTO/TiO_2_-SK/FAPbI_3_ films were calculated by biexponential fitting according to y = y0 + A1*exp(-(x-x0)/*t*_1_) + A2*exp(-(x-x0)/*t*_2_). X-ray diffraction patterns (XRD) were measured by ATX-XRD, Rigaku, Japan in the 2θ range from 4° to 40° at a speed of 10° min^-1^. Top-view and cross-section scanning electron microscopy (SEM) images were characterized by Jeol JSM-6700F, Japan. X-ray photoelectron spectroscopy (XPS) was tested by Thermo Scientific ESCALAB 250Xi.

**Figure S1.** The contact angle images of the TiO_2_ and potassium trifluoromethyl sulfonate modified TiO_2_ (TiO_2_-SK) substrates.

**Figure S2.** (a) The ultraviolet-visible (UV-vis) absorption spectra and (b) Tauc-plot curves of the TiO_2_ and TiO_2_-SK films.

**Figure S3.** The fourier transform infrared spectroscopy (FTIR) tests of the SK, PbI_2_ and SK-PbI_2_ mixtures.

**Figure S4.** X-ray photoelectron spectroscopy (XPS) tests of the SK, perovskite (PVK) and SK modified perovskite (PVK+SK) films.

**Figure S5.** X-ray photoelectron spectroscopy (XPS) tests of the SK, control TiO_2_ and TiO_2_-SK ETLs.

**Figure S6.** (a-b) Top-view and (c-d) cross-sectional scanning electron microscopy (SEM) images of the TiO_2_ and TiO_2_-SK substrates.

**Figure S7.** The ultraviolet photoelectron spectroscopy (UPS) tests of (a) FAPbI_3_, (b) TiO_2_ and TiO_2_-SK films.

**Figure S8.** The Tauc-plot curves of the TiO_2_/FAPbI_3_ and TiO_2_-SK/FAPbI_3_ films.

**Figure S9.** The full width at half maxima (FWHM) of the (a-b) TiO_2_/FAPbI_3_ and (c-d) TiO_2_-SK/FAPbI_3_ perovskite films.

**Figure S10.** The cross-sectional images of the (a) TiO_2_/FAPbI_3_ and (b) TiO_2_-SK/FAPb_3_ perovskite films.

**Figure S11.** The *J-V* curves of the TiO_2_ perovskite solar cells (PSCs) with different SK concentrations.

**Figure S12.** The stability of TiO_2_ and TiO_2_-SK PSCs in N_2_ atmosphere.

**Figure S13.** The stability of TiO_2_ and TiO_2_-SK PSCs in ambient conditions.

**Figure S14.** Cross-comparison of PCE and FF between the published planar TiO_2_ PSCs and this work. ^[1-14]^

**Table S1.** The time-resolved photoluminescence (TRPL) results of glass/FAPbI_3_, FTO/TiO_2_/FAPbI_3_, FTO/TiO_2_-SK/FAPbI_3_ samples.

| Sample | *t*_1_ (ns) | *t*_2_ (ns) |
| --- | --- | --- |
| Glass/FAPbI_3_ | 2199.2 |  |
| FTO/TiO_2_/FAPbI_3_ | 391.9 | 1142.6 |
| FTO/TiO_2_-SK/FAPbI_3_ | 31.6 | 761.7 |

**Table S2.** The electrical impedance spectroscopy (EIS) results of the TiO_2_-FAPbI_3_ and TiO_2_-SK/FAPbI_3_ PSCs.

| Sample | Rtr (Ω) | Rrec (Ω) | CPE (C) |
| --- | --- | --- | --- |
| TiO_2_ PSC | 15.05 | 349 | 7.9*10^^-7^ |
| TiO_2_-SK PSC | 13.78 | 1898 | 3.5*10^^-7^ |

**Table S3.** The photovoltaic performance of PSCs with different SK concentrations.

| Concentration  (mg/mL) | *J*_sc_ (mA cm^-2^) | *V*_oc_ (V) | *FF* | *PCE* (%) |
| --- | --- | --- | --- | --- |
| 0 | 25.4712 | 1130.014 | 0.835139 | 24.013 |
| 0.5 | 25,5211 | 1154.572 | 0.841567 | 24.800 |
| 1.0 | 25.5124 | 1165.066 | 0.848788 | 25.221 |
| 1.5 | 25.4906 | 1167.557 | 0.822017 | 24.452 |

**Reference**

1. H. Huang, P. Cui, Y. Chen, L. Yan, X. Yue, S. Qu, X. Wang, S. Du, B. Liu, Q. Zhang, Z. Lan, Y. Yang, J. Ji, X. Zhao, Y. Li, X. Wang, X. Ding and M. Li, *Joule*, **2022,** 6, 2186-2202.

2. H. Su, J. Zhang, Y. Hu, X. Du, Y. Yang, J. You, L. Gao and S. Liu, *Advanced Energy Materials*, **2021**, 11, 2101454

3. A. Kogo, Y. Sanehira, Y. Numata, M. Ikegami and T. Miyasaka, *ACS Appl Mater Interfaces*, **2018**, 10, 2224-2229.

4. X. Ren, L. Xie, W. B. Kim, D. G. Lee, H. S. Jung and S. Liu, *Solar RRL*, **2019**, 3, 1900176.

5. H. Tan, A. Jain, O. Voznyy, X. Lan, F. P. García de Arquer, J. Z. Fan, R. Quintero-Bermudez, M. Yuan, B. Zhang, Y. Zhao, F. Fan, P. Li, L. N. Quan, Y. Zhao, Z.-H. Lu, Z. Yang, S. Hoogland and E. H. Sargent, **2017**, 355, 722-726.

6. M. Kim, J. Jeong, H. Lu, T. K. Lee, F. T. Eickemeyer, Y. Liu, I. W. Choi, S. J. Choi, Y. Jo, H. B. Kim, S. I. Mo, Y. K. Kim, H. Lee, N. G. An, S. Cho, W. R. Tress, S. M. Zakeeruddin, A. Hagfeldt, J. Y. Kim, M. Gratzel and D. S. Kim, *Science*, **2022**, 375, 302-306.

7. Y. Li, Z. Chen, B. Yu, S. Tan, Y. Cui, H. Wu, Y. Luo, J. Shi, D. Li and Q. Meng, *Joule*, **2022**, 6, 676-689.

8. F. Gao, C. Luo, X. Wang and Q. Zhao, *Small Methods*, **2021**, 5, 5, 2100856.

9. X. Yue, B. Fan, X. Zhao, Y. Yang, S. Qu, Q. Zhang, X. Sun, P. Cui, J. Ma and M. Li, *Sustainable Energy & Fuels*, **2023**, 7, 727-734.

10. Y. Cao, J. Feng, M. Wang, N. Yan, J. Lou, X. Feng, F. Xiao, Y. Liu, D. Qi, Y. Yuan, X. Zhu and S. Liu, *Advanced Energy Materials*, **2023**, 1313, 2302103.

11. K. Li, L. Zhang, Y. Ma, Y. Gao, X. Feng, Q. Li, L. Shang, N. Yuan, J. Ding, A. K. Y. Jen, J. You and S. Liu, *Advanced Materials*, **2023**, 2310651.

12. C. Zhang, A. Baktash, J. A. Steele, D. He, S. Ding, S. Penukula, M. Hao, R. Lin, J. Hou, N. Rolston, M. Lyu, P. Chen, W. Q. Wu and L. Wang, *Advanced Functional Materials*, **2024**, 2315897.

13. H. Guo, G. W. Yoon, Z. J. Li, Y. Yun, S. Lee, Y. H. Seo, N. J. Jeon, G. S. Han and H. S. Jung, *Advanced Energy Materials*, **2023**, 14, 2302743.

14. Y. B. Li, J. K. Cooper, W. J. Liu, C. M. Sutter-Fella, M. Amani, J. W. Beeman, A. Javey, J. W. Ager, Y. Liu, F. M. Toma and I. D. Sharp, *Nature Communications*, **2016**, 7, 12446.
